# A Lateral Flow Immunochromato-graphic Strip Test for Rapid Detection of Oseltamivir Phosphate in Egg and Chicken Meat

**DOI:** 10.1038/s41598-018-35080-5

**Published:** 2018-11-12

**Authors:** Xingdong Yang, Jifei Yang, Yinbiao Wang, Lili Li, Zhongke Sun, Zonghao Yue, Fengshou Tian, Le He, Xiaofei Hu

**Affiliations:** 1 Institute of Food and Drug Inspection, Zhoukou Normal University, Zhoukou, 466001 P. R. China; 2 Key Laboratory of Animal Immunology of the Ministry of Agriculture, Henan Provincial Key Laboratory of Animal Immunology, Henan Academy of Agricultural Sciences, Zhengzhou, 450002 P. R. China; 3 School of Public Health, Xinxiang Medical University, Xinxiang, 453003 P. R. China

## Abstract

A lateral flow immunochromatographic strip test (LFIST) based on a competitive format was developed for rapid and sensitive on-site detection of oseltamivir phosphate (OP) residues in poultry product. The sensitivity (half inhibitory concentration, IC_50_) of the LFIST in the detection of egg and chicken meat samples was confirmed to be 2.56 and 2.63 *µ*g/kg, and the limit detection (LOD) value were 0.43 and 0.42 *µ*g/kg, respectively. For intra-assay and inter-assay reproducibility, recoveries of OP spiked samples ranged between 82.8% and 91.2% with coefficients of variations (CV) less than 5.67% (intra-assay) and 6.52% (inter-assay). The performance of LFIST was comparable to high-performance liquid chromatography (HPLC) in a parallel testing of egg samples and chicken samples. LFIST takes less than 5 minutes, eliminates the dependency on professional personnel, and thus can be used as a surveillance tool for on-site detection of OP residues.

## Introduction

Oseltamivir phosphate (OP) is an ethyl ester prodrug widely used to treat and prevent infections from influenza A and B virus^1,2^. After oral administration, OP is absorbed by the gastrointestinal tract and metabolized into oseltamivir carboxylic acid (OCA) under the action of the liver esterase. OCA is a potent and selective inhibitor of the influenza virus that binds the viral surface protein neuraminidase (NA) and inhibits its activity^3,4^. The specific inhibition of neuraminidase activity finally prevents the release of virus from infected cells and reduces infection-related symptoms^5,6^. In China, the use of OP as an antiviral veterinary drug has been banned in livestock and poultry breeding (Bulletin of the Chinese Ministry of Agriculture under Grant 560)^7^. Nevertheless, OP is still illegally and even excessively used for avian influenza treatment in the poultry industry, especially in chicken farming^8,9^. OP residues may enter the human body via the food chain and affect human health. Researches showed that exposure to excessive OP can lead to gastrointestinal disturbances^10,11^, abnormal neuropsychiatric symptoms^12,13^, and sudden deaths^14,15^. Therefore, the detection of OP in agriculture and food supply is essential for human health and a surveillance system for monitoring OP residues is required.

To date, several analytical methods for the detection of OP have been reported, including HPLC^16,17^, ultra-high- performance liquid chromatography-tandem mass spectrometry (UPLC–MS/MS)^18,19^, and liquid chromatography-mass spectrometry(LC-MS)^20^. Although these methods are highly accurate and sensitive, they are dependent on expensive analytical instruments, complex pre-treatment steps, and skilled professionals. Moreover, they are not suitable for high-throughput assay and real time on-site detection. On the contrary, LFIST, which utilizes the specific interaction between antibody and antigen in a sandwich format^21^ or competitive format^22^, is rapid and easy to be used by non-professionals. LFISTs for the specific detection of bacteria^23^, viruses^24^, pesticides^25^, veterinary drugs^26–28^, toxins^29,30^ and allergens^31,32^ have been developed and tested. However, there is no available LFIST for detecting OP currently. Hence, in this study, we prepared a highly specific monoclonal antibodies (mAbs) against OP and developed a LFIST using colloidal gold labelled mAbs as the probe. The LFIST was evaluated in term of sensitivity, specificity, and accuracy by testing OP spiked egg samples and chicken samples. It showed that the LFIST was OP-specific and could provide qualitative results with naked eyes within 5 min and quantitative results with the aid of a strip reader.

## Materials and Methods

### Reagents, Materials, and Apparatus

OP and OCA were bought from Sigma (St. Louis, MO, USA). 1-(3-(dimethylamino) propyl)-3-ethylcarbodiimide hydrochloricde (EDC) and N-Hydroxy succinimide (NHS) were purchased from Fluka, China. Bovine serum albumin (BSA), ovalbumin (OVA), Freund’s complete adjuvant (FCA) and Freund’s incomplete adjuvant (FIA) were obtained from BDH (VWR International Ltd.). 4-bromobutyric acid ethyl ester and *N*,*N*-dimethylformamide (DMF) were bought from Aladdin Chemistry Ltd. (Shanghai, China). BALB/c mice were acquired from the SPF standard Laboratory Animal Center (Zhengzhou University, China) and raised according to the animal Ethics committee of Zhoukou Normal University. All reagents and solvents were of analytical grade or higher in this study.

Thermo Scientific NaoDrop 2000c UV scanner, gel imaging system, and the Multifuge X1R high-speed refrigerated centrifuge were bought from Thermo Scientific (USA). The XYZ Biostrip Dispenser, CM4000 Cutter, and TSR3000 membrane strip reader were purchased from Bio-Dot (Richmond, CA).

### Synthesis of Artificial Antigens

Chemical modification of OCA was carried out using the active ester method to prepare OP-BSA and OP-OVA conjugates according to the instruction described by Liu and coauthors with minor modification^33^, Briefly, 58.0 mg of OCA was mixed with 26.0 mg of NHS, 42.0 mg of EDC and 2 mL of DMF for 6 h at room temperature (RT). Then, the mixture was added into 3.0 mL of PBS dissolving 107.0 mg of BSA and stirred for 12 hours at 4 °C. The above solution was dialyzed against PBS, collected by centrifugation at 1510 × g for 20 min at 4 °C, and then stored at −20 °C. UV scanning was employed to deduce the conjugation ratio of OCA with BSA and OVA.

### Preparation of mAbs against OP

Hybridomas secreting anti-OP mAbs were generated according to previously described^34^. Each BALB/c female mice was subcutaneously immunized with 60 *µ*g OP-BSA (immunogen) in 100 *µ*L immunoadjuvant (the first time with FCA, the rest with FIA) at intervals of 21 days for four times. Serum antibodies and mAbs were collected and detected by indirect and competitive indirect ELISAs. Ascites fluids were produced in paraffin-primed BALB/c mice and IgG was purified using a HiTrap protein A column. Affinity and isotype of the mAbs were characterized using ELISA and a mouse mAbs isotyping kit (Sigma, USA).

### Indirect ELISA and Competitive Indirect ELISA

Indirect ELISA was performed as following: 96-well microplates were coated with OP-OVA (coating antigen) in 0.05 M carbonate-bicarbonate buffer (pH 9.6) by incubation at 4 °C overnight. Then the wells were blocked with 5% skimmed milk at 37 °C for 1 h. Two-fold serially diluted serum sample or cell culture supernatant was added and incubated at 37 °C for 30 min. Goat-anti-mouse IgG-HRP conjugate was added and incubated for another 30 min at 37 °C. The optical densities (ODs) were measured at 450 nm with a microplate reader after color development with TMB (3,3′, 5,5;-tetramethylbenzidine) chromogen solution and termination with 2 mol/L H_2_SO_4_. The competitive indirect ELISA was carried out as following: microplates were coated as described above, and serially diluted free OP in PBS was then added together with the antibody and incubated for 30 min at 37 °C. The following procedures were identical to those employed in indirect ELISA. During the steps, TBST [TBS containing 0.05% Tween 20 (v/v), pH 7.4] was used as wash buffer to remove unbound antigens or antibodies.

### Assembly of the LFIST

**Figure 1:**
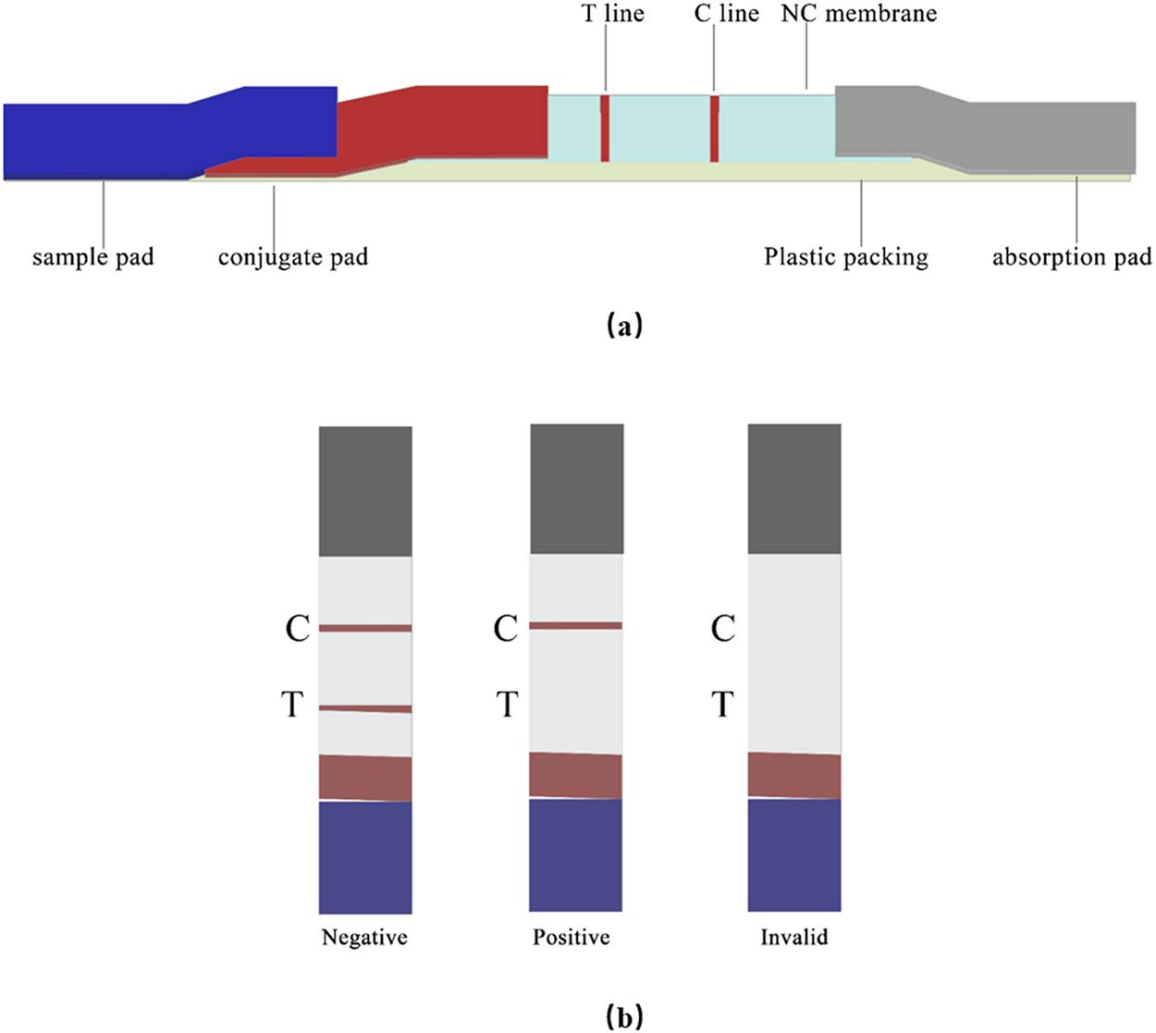
**(a)** Structure diagram of the (LFIST). **(b)** The visual results determination in test procedure.

Gold was prepared by gold chloride of reduction with 1% sodium citrate (w/v), sodium carbonate (0.2 mol/L) was used to adjust the PH of colloidal gold solution to 8.2. Anti-OP mAbs solution (50 *µ*L) was 2-fold serially diluted in DDW (double distilled water) mixed with 200 *µ*L of colloidal gold solution, and then 200 *µ*L of a 10% sodium chloride solution was added at room temperature to identify the optimum proportion of colloidal gold solution and mAbs solution. After 8 minutes, with the concentration of mAbs decreased, the color of solution changed from brilliant red to blue. The most appropriate concentration of colloidal gold labeling mAbs was the lowest concentration of mAbs solution which color had changed. The treated sample pad, conjugate pad, absorption pad, and NC membrane were assembled according to the previously described steps^34^ (Fig. 1a).

### Principle of the LFIST

Colloidal gold was prepared and labelled with anti-OP mAbs according to previously described^35^. Colloidal gold labelled mAbs, OP-BSA conjugate, goat-anti-mouse IgG were respectively dispensed onto the conjugate pad, the test line (T line), and the control line. The pre-treated sample pad, conjugate pad, absorption pad, and NC membrane were assembled according to the previously described steps^36^. After loading onto the sample pad, the sample solution migrates to the absorbent pad in less than 5 min. If the sample contains OP, free OP in the sample will compete with the OP-BSA on T line for binding gold-labeled mAbs, interfering color developing on the T line. Thus, the concentration of OP in the sample is inversely correlated with the intensity of color on the T line. The control line will always appear as a visible red line, indicating the validation of the test. The test is considered as invalid if no control line appear, and should be done again using a new test strip (Fig. 1b).

### Sample Pretreatment for the LFIST

Chicken and egg samples from market were pretreated and tested for the presence of OP by UPLC–MS/MS. Three gram of sample was homogenized in 9 mL of 0.25 mol/L ammonium acetate buffer solution, added 9 ml of a mixture of methanol and acetonitrile (2:1, v/v), vibrated for 12 min, and centrifugated at 3900 × g for 5 min. 2000 *µ*L of the supernatant was evaporated to dryness at 45 °C under a gentle flow of nitrogen gas. The residue was then resuspended in 1000 *µ*L of the mixed solution of PBS and methanol (4:1, V/V), and tested.

### Evaluation of the Performance of LFIST

The performance of LFIST was evaluation in terms of sensitivity, specificity, and accuracy. The sensitivity of the LFIST was determined by detection of negative chicken samples spiked with 0.0, 1.0, 2.0, 4.0, 8.0, 16.0, 32.0, 64.0, 128.0, and 256.0 ng/mL of OP. Each sample was tested in triplicate. To obtain the relative optical density (ROD) for constructing a standard curve, color density of the T line was read by a TSR3000 membrane strip reader (Bio-Dot). IC_50_ was obtained from the linear regression equation.

The specificity of the LFIST was identified by testing its cross-reactivity (CR) with similar competitors to OP including amantadine, moroxydine, Ribavirin, acyclovir, amoxicillin trihydrate, and enrofloxacin. OP, OCA, or the competitors were added to the negative egg samples at a concentration of 1 *µ*g/mL respectively, and tested by LFIST, and the CR were calculated by the equation: CR (%) = (IC_50_ of OP)/IC_50_ of the other competitors) × 100%.

To estimate the accuracy, chicken samples containing 5, 10, and 20 *µ*g/kg of OP were tested by LFIST using the same batch of strips (n = 6). For inter-assay precision, three batches of strips were used in LFIST. Accuracy and precision were indicated as recovery and CV, respectively.

### Authenticity of LFIST

Egg and chicken samples, containing three different OP concentrations (3.5, 9.7, and 27.9 *µ*g/kg, representing low, medium and high levels of the residues), were detected by LFIST and HPLC, respectively. Results from two methods were compared to the given concentrations by one-sample T test respectively and analyzed by independent-sample T test. The differences between the test values and the relative given concentration were considered statistically significant at P < 0.05.

## Results

### Preparation of Artificial Antigens and mAbs

**Figure 2:**
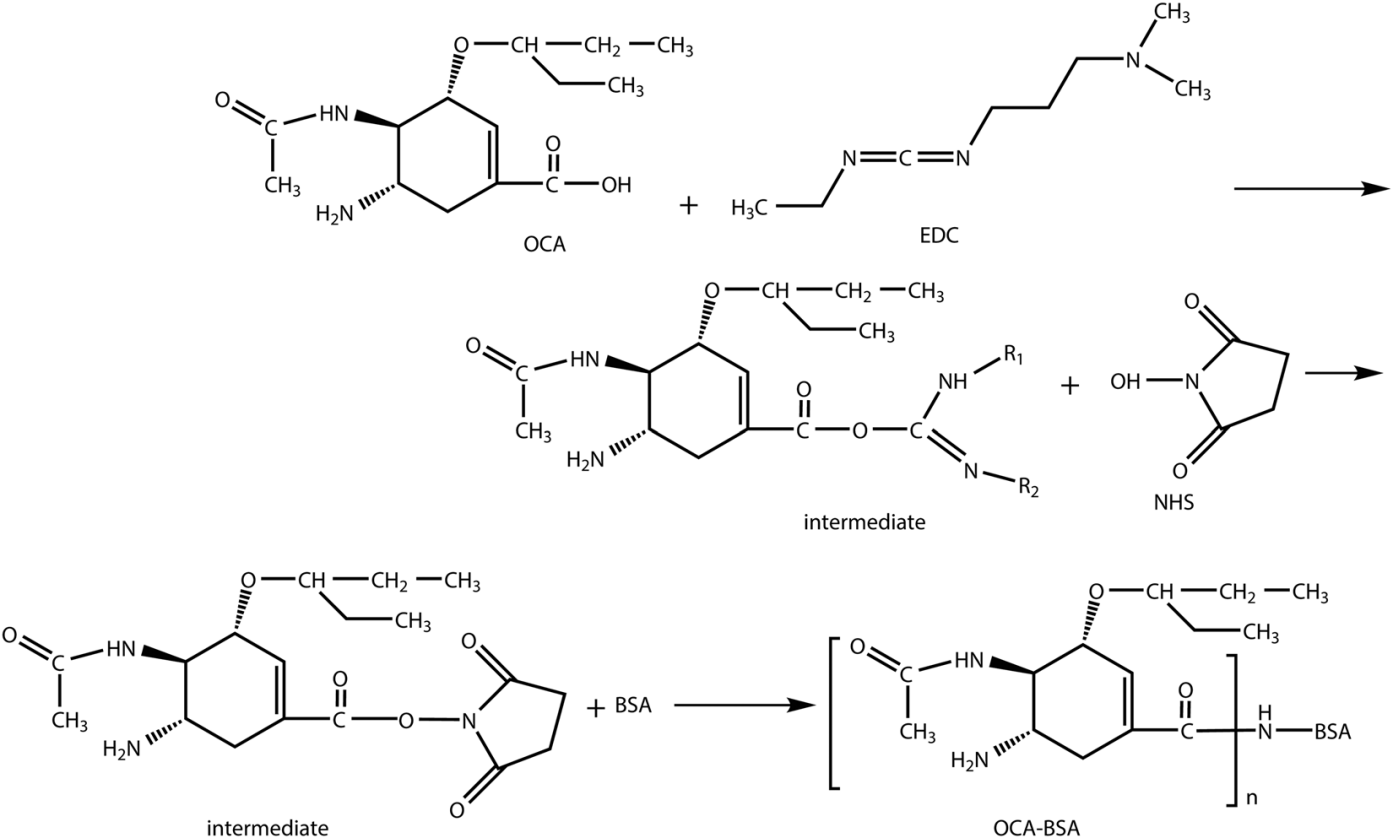
Chemical strutures of the synthetic routes to OP-BSA via the active ester method.

The immunogen (OP-BSA) and coating antigen (OP-OVA) in ELISAs were synthesized by coupling free amino groups of the carrier proteins and carboxyl acid group of the OCA using the active ester method (Fig. 2). The conjugation ratio for OP-BSA and OP-OVA were about 18.1:1 and 16.4:1 respectively. After cell fusion and screening with ELISAs, anti-OP mAbs 1D7, 2B3, 2E6, and 4G5 were obtained, and IgG for each mAbs was purified from ascites fluid using protein A column. The titres of 1D7, 2B3, 2E6, and 4G5 were 1:2.56 × 10^5^, 1:1.28 × 10^5^, 1:5.12 × 10^5^ and 1:2.56 × 10^5^, respectively; the affinity constants (Ka) of 1D7, 2B3, 2E6, and 4G5 were 7.96 × 10^9^, 3.24 × 10^9^, 1.80 × 10^10^, and 8.15 × 10^9^ L/mol, respectively. mAbs 2E6 (IgG1) showed the best IC_50_ value of 2.35 ng/mL and was selected to be used in the development of LFIST.

### Sensitivity of the LFIST

**Figure 3:**
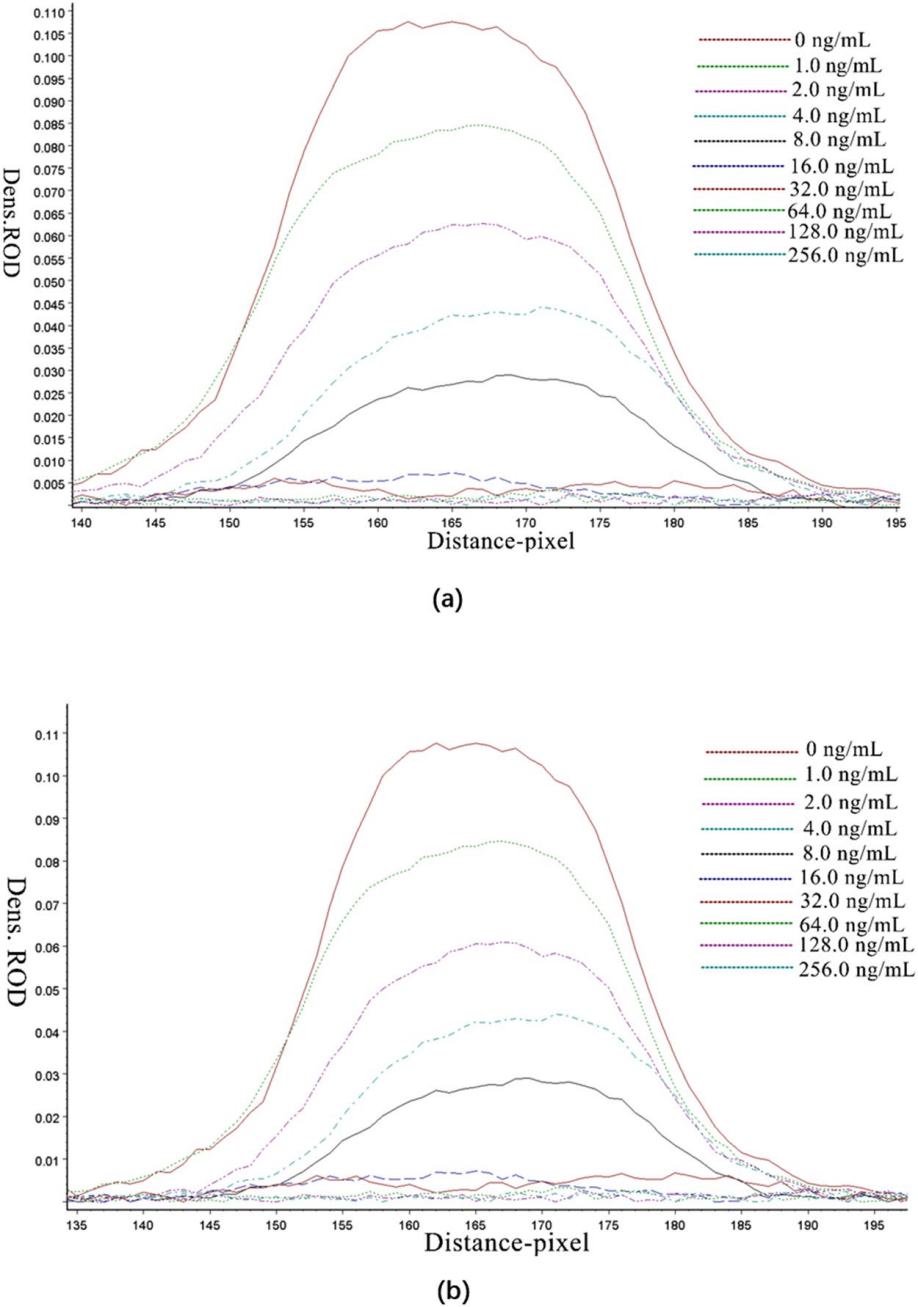
ROD curves of the egg sample extracts **(a)** and chicken sample extracts **(b)**. The matrix extracts from negative samples spiked with 0.0, 1.0, 2.0, 4.0, 8.0, 16.0, 32.0, 64.0, 128.0, and 256.0 ng/mL of OP were tested by LFIST and T lines were scanned with a TSR3000 membrane strip reader.

Sensitivity was determined by testing the responses of LFIST to detect a series of OP spiked samples. Relative optical density (ROD) of the T lines were scanned by a Bio-Dot TSR3000 membrane strip reader. ROD values and OP concentrations in the samples had an inverse relationship as shown for detecting egg samples (Fig. 3a and Table 1) and chicken samples (Fig. 3b and Table 2). In both detections, the G/D × A (area) and G/peak of the ROD decreased with the increase of OP concentration in the samples.

**Table 1:** G/Peak and G/D × area of the ROD of T lines from egg samples spiked with OP.

OP concentrations (µg/Kg)	G/D × area-ROD (pixel)	G/Peak-ROD (pixel)
0	185.7710	0.1081
1	133.3474	0.0855
2	103.9262	0.0612
4	74.1141	0.0445
8	47.2295	0.0291
16	19.1198	0.0122
32	2.4209	0.0070
64	2.2358	0.0035
128	2.1082	0.0029
256	2.0447	0.0027

**Table 2:** G/Peak and G/D × area of the ROD of T lines from chicken samples spiked with OP.

OP concentrations (µg/Kg)	G/D × area-ROD (pixel)	G/peak-ROD (pixel)
0	184.8360	0.1077
1	130.7277	0.0846
2	102.5247	0.0609
4	73.1491	0.0440
8	46.1188	0.0290
16	19.1297	0.0118
32	2.3543	0.0068
64	2.1369	0.0033
128	2.0315	0.0027
256	1.9792	0.0026

**Figure 4:**
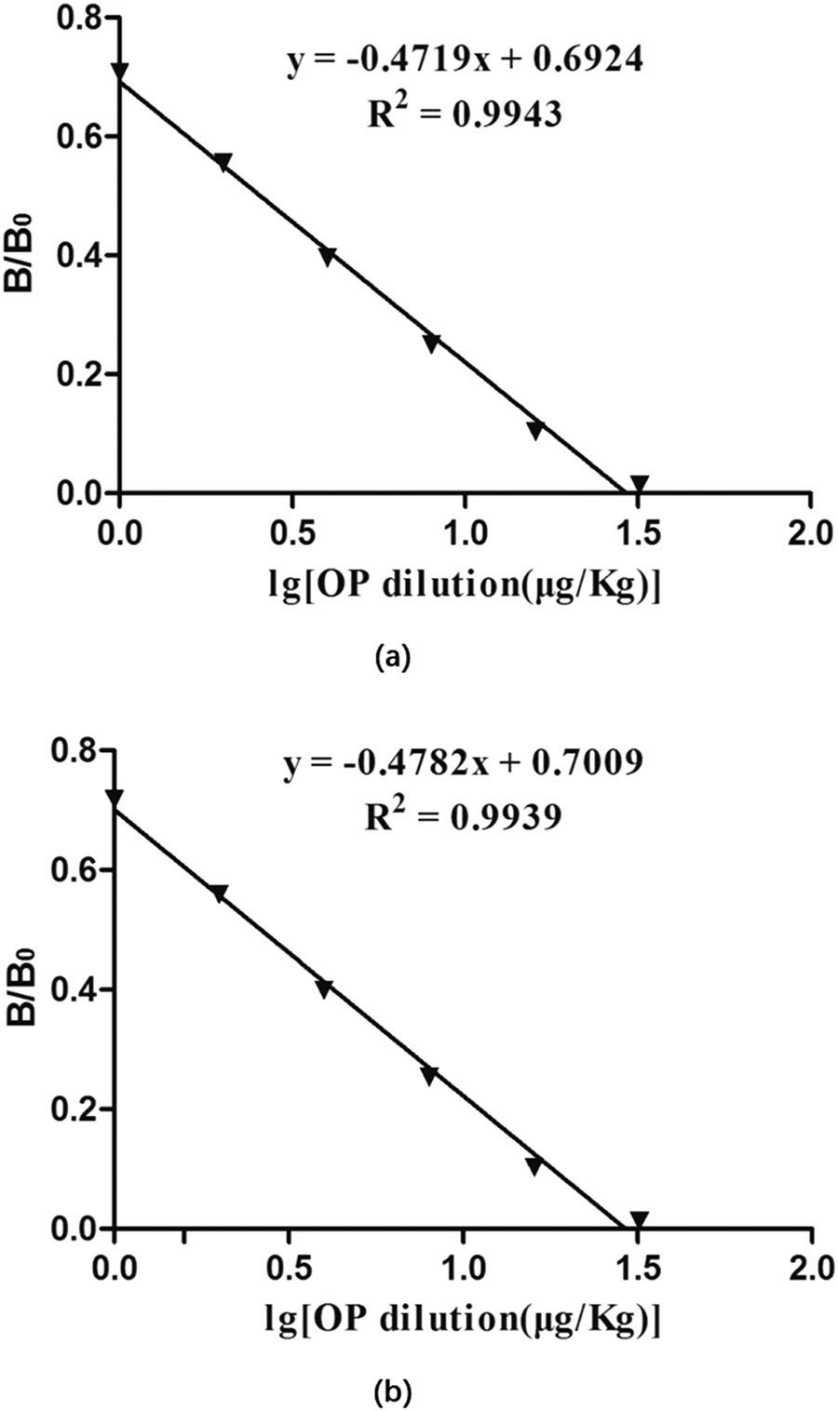
Standard curves for detecting egg samples **(a)** and chicken meat samples **(b)** using LFIST. The X-axis is shown as the logarithmic concentrations of OP. B/B_0_ expresses the percentage of ROD of the spiked samples divided by ROD of the negative samples. IC_50_ for testing each sample were calculated as 2.56 ng/mL **(a)** and 2.63 ng/mL **(b)**, respectively.

**Figure 5:**
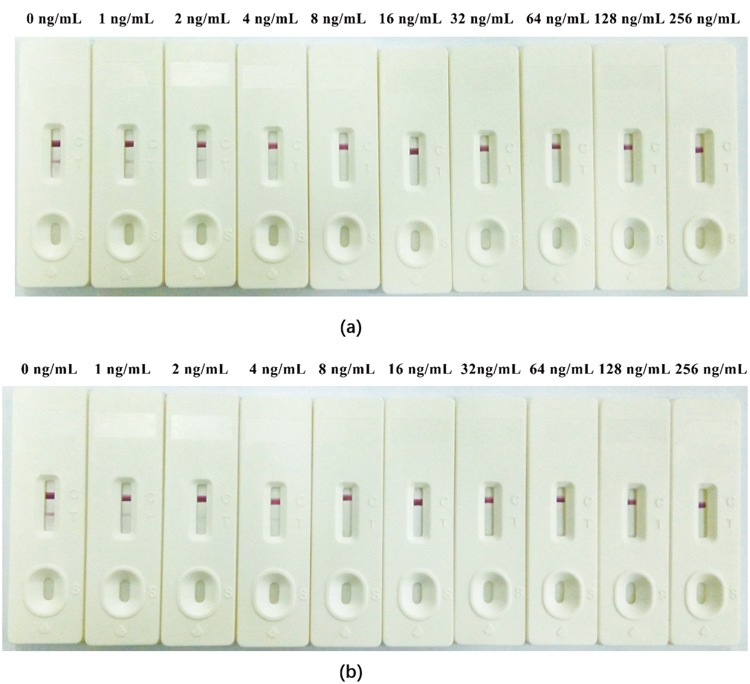
Qualitative detections of OP in egg samples (**a**) and chicken samples (**b**). A serial dilution (0 to 256 ng/mL) of OP was added into the samples and tested by the LFIST. The results were judged with naked eye based on color differences in the T lines.

The concentration of standard OP and the G/D × area ROD showed a clear linear association within the range of 1–32 ng/mL for egg samples (Fig. 4a) and chicken samples (Fig. 4b). A quantitative calibration curve was drawn by plotting B/B_0_ percentage of the G/D × area-ROD obtained from the standard samples against the logarithmic concentrations of OP in egg samples or in chicken samples. Based on the regression equations, the calculated IC_50_ were 2.56 ng/mL (R^2^ = 0.9943) for testing egg samples and 2.63 ng/mL (R^2^ = 0.9939) for testing chicken samples. The LOD of LFIST was quantitatively defined as the amount of OP in the standard sample solution that caused a 20% decrease of the G/peak-ROD compared with that produced by the blank sample. The LODs were determined to be 0.43 *μ*g/kg for testing egg samples and 0.42 *μ*g/kg for testing chicken samples. In qualitative testing with naked eyes, the LOD was determined by the minimal amount of OP that produced a clearly visible difference between the T lines for detecting standard samples and negative samples. The LODs in testing egg samples (Fig. 5a) and chicken samples (Fig. 5b) both reached 4 *μ*g/kg by unaided visual assessment.

### CR of the LFIST

To identify the specificity of the test, LFIST was used to detect egg samples spiked with OP and other competitors including amantadine, moroxydine, ribavirin, acyclovir, amoxicillin trihydrate, and enrofloxacin doped at a concentration of 1000 *µ*g/kg. It was found that the LFIST gave a 100% CR with OP and oseltamivir carboxylic acid, and less than 0.09% CR with other competitors (Table 3). Thus, LFIST for the detection of OP was highly specific and expressed negligible CR with amantadine, moroxydine, ribavirin, acyclovir, amoxicillin trihydrate, and enrofloxacin.

**Table 3:** CR of the LFIST.

Compounds tested	IC_50_ (µg/Kg)	CR (%)
Oseltamivir Phosphate	2.56	100
Oseltamivir carboxylic acid	2.56	100
Amantadine	>256	<0.09
Moroxydine	>3.0 × 10^3^	<0.09
Ribavirin	>3.0 × 10^3^	<0.09
Acyclovir	>3.0 × 10^3^	<0.09
Amoxicillin trihydrate	>3.0 × 10^3^	<0.09
Enrofloxacin	>3.0 × 10^3^	<0.09

### Recovery of OP in Egg and Chicken Samples

In order to determine the accuracy of the LFIST, egg and chicken samples containing 5, 10, and 20 *μ*g/kg of OP were tested for recovery efficiency. For intraassay recovery, the recoveries ranged from 84.6% to 91.2% with the highest CV at 5.67%. For interassay recovery, the recoveries ranged from 82.8 to 90.6% with the highest CV at 6.52% (Table 4).

**Table 4:** Recovery and precision of LFIST for detecting OP spiked in egg and chicken samples.

Spiked OP (µg/kg)	Intraassay			Interassay		
	Mean ± SD (µg/kg)	Recovery (%)	CV (%)	Mean ± SD (µg/kg)	Recovery (%)	CV (%)
**Egg samples**						
5	4.32 ± 0.20	86.4 ± 4.0	4.63	4.38 ± 0.27	87.6 ± 5.4	6.19
10	8.93 ± 0.28	89.3 ± 2.8	3.14	8.67 ± 0.44	86.7 ± 4.4	5.07
20	18.24 ± 0.69	91.2 ± 3.5	3.78	18.09 ± 0.87	90.5 ± 4.3	4.81
**Chicken samples**						
5	4.23 ± 0.24	84.6 ± 4.8	5.67	4.14 ± 0.27	82.8 ± 5.4	6.52
10	8.73 ± 0.35	87.3 ± 3.5	4.01	8.89 ± 0.50	88.9 ± 5.0	5.62
20	18.11 ± 0.77	90.55 ± 3.9	4.25	18.17 ± 0.86	90.6 ± 4.3 4.73	

Egg samples and chicken samples spiked with OP at 5, 10, and 20 *μ*g/kg were respectively tested using LFIST. Intraassay precision was evaluated using the same batch of strips in LFIST (n = 6). For interassay precision, three batches of strips were used in LFIST. The recoveries and CV were calculated from triplicate assays in all cases.

### Authenticity of LFIST

A comparison between LFIST and HPLC was performed by detecting three different concentration levels of OP (3.5, 9.7, and 27.9 *µ*g/kg). There was no significant difference between the two methods for detecting both egg samples and chicken samples (P > 0.05) as shown in Table 5.

**Table 5:** Comparison of LFIST with HPLC for detecting three levels of OP residues in egg and chicken samples.

OP concentrations (µg/kg)	LFIST (µg/kg)	HPLC (µg/kg)
**Egg samples**		
3.5	3.30 ± 0.15	3.34 ± 0.12
9.7	9.14 ± 0.36	9.29 ± 0.34
27.9	26.72 ± 0.73	26.98 ± 0.53
**Chicken samples**		
3.5	3.28 ± 0.17	3.31 ± 0.13
9.7	8.98 ± 0.47	9.22 ± 0.29
27.9	26.47 ± 0.72	26.66 ± 0.65

## Discussion

Although the use of OP as a veterinary drug has been banned, illegal and even excessive use occurs in cases, which poses a threat to public health in developing countries. Customers suffer vomiting, nausea, diarrhea, and abnormal neuropsychiatric symptoms from eating animal-derived food products contaminated with OP. Thus, it is highly desired to develop an easy-to-use diagnostic for detecting OP in food products to strengthen surveillance. Although HPLC, UPLC–MS/MS and LC-MS are highly accurate and sensitive, they rely on expensive analytical instruments and trained personnel, and cannot fulfill the need to detect OP at the point-of-care. On the contrary, LFIST, which is an immunoassay dependent on the interaction between antibody and antigen, is a one-step chromatographic assay that can be performed by customers in the market or at home. Hence, we developed a LFIST for the detection of OP for the first time. The LFIST integrated all the required reagents in a test strip and took less than 5 min to give the result, which could be judged qualitatively with naked eyes or quantitatively with a strip reader.

The key to a successful LFIST is the preparation of monoclonal antibodies with strong specificity and high affinity. The immunogenicity of antigens is crucial to activate the immune system and then stimulate the production of antibodies. As a small molecule drug with a molecular weight of 312.35, OP is a hapten which cannot stimulate the host to produce antibodies. The conjugation of OP with carrier proteins such BSA, OVA, and KLH to form a complete antigen is an essential step for the induction of antibodies to OP after immunization^37^. In this study, the active metabolite of OP, OCA was coupled with BSA and OVA through the active ester method to prepare the immunogen and coating antigen in ELISAs. The conjugation ratio for OP-BSA and OP-OVA were about 18.1:1 and 16.4:1 respectively. Anti-OP mAbs 2E6, which showed an affinity constant (Ka) of 3.24 × 10^9^ L/mol and an IC_50_ value of 2.35 ng/mL was screened by indirect and competitive indirect ELISA, and selected for conjugation with colloidal gold to be used as a probe in the development of LFIST.

The LFIST was evaluated in terms of sensitivity, specificity, and recovery by testing OP residues in egg samples and chicken samples. IC_50_ of the LFIST were confirmed to be 2.56 *µ*g/kg and 2.63 *µ*g/kg in the detection of egg samples and chicken samples respectively, indicating that it was highly sensitive. The LOD were 0.43 *µ*g/kg and 0.42 *µ*g/kg for detecting OP in egg samples and chicken samples respectively, which is sufficient for the detection of OP in these samples. For intra-assay and inter-assay reproducibility, recoveries of OP spiked samples ranged from 84.6% to 91.2% for testing egg samples, and 82.8% to 90.6% for testing chicken samples. CV were 3.14–5.67% and 4.73–6.52% for detecting egg and chicken samples, respectively. No significant difference was found in a parallel testing of egg and chicken samples by HPLC and LFIST.

In summary, we developed a sensitive and specific LFIST for rapid detection of OP residues in chicken and eggs. The LFIST is capable to provide both qualitative and semi-quantitative determination of OP at the point-of-care which makes it a very convenient tool to monitor food safety.

### Ethical statement

All mice in the experiment were approved by the animal Ethics committee of Zhoukou Normal University (Approval No. ZKNU-1-2017041601-1001) and in accordance with all applicable institutional and governmental regulations concerning the ethical use of animals. This article does not contain any studies with human participants performed by any of the authors.

### Statistics

Te mean ± standard deviation (SD) was calculated and all data were plotted using GraphPad Prism 5.0 (Graphpad, La Jolla, CA, USA).
